# Antiprotozoal Activity of Benzoylthiourea Derivatives against *Trypanosoma cruzi*: Insights into Mechanism of Action

**DOI:** 10.3390/pathogens12081012

**Published:** 2023-08-03

**Authors:** Patrícia Morais Lopes Pereira, Bruna Terci Fernandes, Vitória Ribeiro dos Santos, Weslei Roberto Correia Cabral, Maria Isabel Lovo-Martins, Lais Alonso, César Armando Contreras Lancheros, Jéssica Carreira de Paula, Priscila Goes Camargo, Helena Tiemi Suzukawa, Antônio Alonso, Fernando Macedo, Celso Vataru Nakamura, Eliandro Reis Tavares, Marcelle de Lima Ferreira Bispo, Lucy Megumi Yamauchi, Phileno Pinge-Filho, Sueli Fumie Yamada-Ogatta

**Affiliations:** 1 Graduate Program in Microbiology, Department of Microbiology, State University of Londrina, Londrina 86057-970, Brazil; pml.pereira@hotmail.com (P.M.L.P.); terci.bruna@gmail.com (B.T.F.); wesleircc@gmail.com (W.R.C.C.); tiemi.imeit@gmail.com (H.T.S.); pingefilho@uel.br (P.P.-F.); 2 Laboratory of Molecular Biology of Microorganisms, Department of Microbiology, State University of Londrina, Londrina 86057-970, Brazil; vitoria.ribeiro002002@gmail.com (V.R.d.S.); tavares.eliandro@uel.br (E.R.T.); 3 Laboratory of Experimental Immunopathology, Department of Immunology, Parasitology and General Pathology, State University of Londrina, Londrina 86057-970, Brazil; misabel@uel.br; 4 Institute of Physics, Federal University of Goiás, Goiania 74690-900, Brazil; laisalonso2@hotmail.com (L.A.); alonso@ufg.br (A.A.); 5 Center for Human, Biological, Social and Educational Sciences, State University of Paraná, Paranagua 83203-560, Brazil; cesarlancheros@gmail.com; 6 Department of Parasitology, University of Granada, 18071 Granada, Spain; jessicacarreira123@gmail.com; 7 Laboratory of Medicinal Molecules Synthesis, Department of Chemistry, State University of Londrina, Londrina 86057-970, Brazil; priscilagcmg@gmail.com (P.G.C.); mlfbispo@uel.br (M.d.L.F.B.); 8 Laboratory of Technological Innovation in the Development of Drugs and Cosmetics, Department of Basic Health Sciences, State University of Maringá, Maringa 87020-900, Brazil; cvnakamura@uem.br

**Keywords:** autophagy-dependent pathway, cell death, Chagas disease, epimastigote forms, mechanism of action, ultrastructural changes

## Abstract

For decades, only two nitroheterocyclic drugs have been used as therapeutic agents for Chagas disease. However, these drugs present limited effectiveness during the chronic phase, possess unfavorable pharmacokinetic properties, and induce severe adverse effects, resulting in low treatment adherence. A previous study reported that *N*-(cyclohexylcarbamothioyl) benzamide (**BTU-1**), *N*-(*tert*-butylcarbamothioyl) benzamide (**BTU-2**), and (4-bromo-*N*-(3-nitrophenyl) carbamothioyl benzamide (**BTU-3**) present selective antiprotozoal activity against all developmental forms of *Trypanosoma cruzi* Y strain. In this study, we investigated the mechanism of action of these compounds through microscopy and biochemical analyses. Transmission electron microscopy analysis showed nuclear disorganization, changes in the plasma membrane with the appearance of blebs and extracellular arrangements, intense vacuolization, mitochondrial swelling, and formation of myelin-like structures. Biochemical results showed changes in the mitochondrial membrane potential, reactive oxygen species content, lipid peroxidation, and plasma membrane fluidity. In addition, the formation of autophagic vacuoles was observed. These findings indicate that **BTU-1**, **BTU-2,** and **BTU-3** induced profound morphological, ultrastructural, and biochemical alterations in epimastigote forms, triggering an autophagic-dependent cell death pathway.

## 1. Introduction

Chagas disease (CD) is a potentially life-threatening neglected tropical disease (NTD) caused by the protozoan *Trypanosoma cruzi*. It is estimated that 6 to 7 million people are infected with the protozoan in the world, most of them in Latin American countries where CD is endemic [1]. However, due to intense population mobility and immigration of people from the endemic regions, more cases have been detected in non-endemic areas, such as Canada, the United States of America, Europe, Africa, and the Western Pacific [2].

Vector-borne transmission is the classic route of infection by *T. cruzi* in mammalian hosts. About 150 species of blood-sucking bugs of the Triatominae subfamily can transmit the parasite through their feces and/or urine [3]. Additionally, the protozoan can be transmitted by oral or congenital routes, blood transfusion, and organ transplantation [1,4]. Clinically, CD comprises an acute phase characterized by high parasitemia and tissue parasitism. Most patients remain asymptomatic, but some may experience non-specific symptoms, such as fever, malaise, hepatosplenomegaly, and atypical lymphocytosis. About 1 to 5% of infected individuals may have cardiac involvement and fatal outcome. Untreated individuals develop a long-lasting chronic phase, with the majority of patients presenting the indeterminate form of the disease (characterized *T. cruzi* seropositivity and absence of symptoms). However, after several decades of acute infection, approximately 30 to 40% of individuals may progress to the symptomatic chronic phase with organ involvement, including cardiomyopathy, mega viscera (megaesophagus and/or megacolon), and neurological disorders [4].

Despite significant efforts to develop vaccines against *T. cruzi* infection with promising experimental results, no vaccine is currently available for CD [5]. Therefore, etiological treatment remains the only therapeutic strategy for controlling CD, which has been carried out for decades with two nitroheterocycle drugs, benznidazole (BZN) and nifurtimox (NFX) [6]. Both drugs have a trypanocidal effect and significantly reduce parasitemia during the acute and early chronic phases, but their efficacy drastically declines during the late chronic phase. Moreover, they require prolonged treatment and can induce several side effects, including skin rash, digestive intolerance, and mutagenic and genotoxic potential, frequently leading to treatment discontinuation [7]. In addition, the emergence of *T. cruzi* strains resistant to both drugs [8,9] and the presence of dormant amastigotes during drug treatment [10] further complicate CD therapy. Consequently, there is an urgent need to develop new safe, effective, and affordable drugs for CD treatment.

In this context, thiourea is a relevant class of sulfur-bearing substances with notable synthetic and biological versatility. Thiourea derivatives have shown promising pharmaceutical potential due to their diverse pharmacological activities, including antitumoral [11], antioxidant [12], antidiabetic [13], and antimicrobial [14,15,16,17,18,19,20] properties. Notably, the thiourea moiety has been identified in several compounds with potential for the development of new therapeutic compounds to treat diseases caused by trypanosomatid species [21,22,23].

In a previous study, we assessed the antiprotozoal activity of sixteen benzoylthiourea derivatives against *T. cruzi* Y strain [23]. Among them, *N*-(cyclohexylcarbamothioyl) benzamide (**BTU-1**), *N*-(*tert*-butylcarbamothioyl) benzamide (**BTU-2**), and 4-bromo-*N*-(3-nitrophenyl) carbamothioyl benzamide (**BTU-3**) exhibited selective antiprotozoal activity against *T. cruzi*, inhibiting the proliferation of epimastigotes and amastigotes, as well as the viability of trypomastigotes, in non-toxic concentrations to mammalian cells [23]. In this study, we further investigated the antiprotozoal activity of **BTU-1**, **BTU-2**, and **BTU-3** against *T. cruzi* Y strain and suggested a potential mechanism of action involved in parasite death. We focused on epimastigotes, although they were not found in the mammalian host, and these developmental forms are easily cultured in vitro [24].

## 2. Materials and Methods

### 2.1. Benzoylthiourea Derivatives

The compounds *N*-(cyclohexylcarbamothioyl) benzamide (**BTU-1**), *N*-(*tert*-butylcarbamothioyl) benzamide (**BTU-2**), and 4-bromo-*N*-(3-nitrophenyl) carbamothioyl benzamide derivative (**BTU-3**) were previously synthesized and fully characterized by spectroscopic and spectrometric methods [17] at the Laboratory of Synthesis of Medicinal Molecules, Universidade Estadual de Londrina, Londrina, Paraná, Brazil. A stock solution of the compounds was prepared in 10% dimethylsulfoxide (DMSO *v/v*, Sigma-Aldrich^®^, Barueri, Brazil) and then diluted in the culture medium. The DMSO concentration in the assays did not exceed 1%. Tests containing only the medium or medium plus 1% DMSO were used as an untreated control in all assays. The **BTU** concentrations causing 50% (IC_50_: **BTU-1** = 26.4; **BTU-2** = 13.4; and **BTU-3** = 61.1 µM) and 90% (IC_90_: **BTU-1** = 81.65; **BTU-2** = 48.37; and **BTU-3** = 264.9 µM) growth inhibition after 72 h were previously determined [23].

### 2.2. Parasite

Epimastigotes of *T. cruzi* Y strain were maintained by weekly transfers in liver infusion tryptose (LIT) medium, pH 7.4 [24], supplemented with 10% heat-inactivated fetal bovine serum (FBS) at 28 °C. For all assays, epimastigotes were harvested from four-day incubation cultures, and a cell density of 1.0 × 10^6^ cells/mL was used, unless otherwise specified.

### 2.3. Time-Kill Kinetics

Epimastigotes were inoculated into a 24-well plate containing LIT+FBS, along with **BTU-1** (26.4 µM), **BTU-2** (13.4 µM) or **BTU-3** (61.1 µM), which correspond to the IC_50_ values after 72 h (IC_50/72 h_) of incubation, determined previously [23]. Cell growth was estimated by direct counting using a hemocytometer (Improved Double Neubauer) every 24 h, up to a period of 96 h. Wells containing growth medium alone or growth medium plus 1% DMSO served as controls. The means cell counts were plotted as the percentage of cell number compared to untreated control vs. time (h). Only motile parasites exhibiting typical morphology were counted. Additionally, the effect of **BTU**s on metabolic activity was analyzed using resazurin (100 µM, Merck, Barueri, Brazil) as described by Rolón et al. [25].

### 2.4. Studies on the Mechanism of Action

Epimastigotes were incubated with **BTU-1** (26.4 µM), **BTU-2** (13.4 µM), or **BTU**-3 (61.1 µM) in all assays at 28 °C, unless otherwise specified. Afterward, the cells were washed twice with 0.15 M phosphate-buffered saline, pH 7.2 (PBS), and were processed as specified for each assay. Untreated epimastigotes were used as the control in all assays.

#### 2.4.1. Transmission Electron Microscopy (TEM) Analysis

Epimastigotes were treated with the IC_50/72 h_ and IC_90/72 h_ of the compounds for 72 h [23]. After this period, the cells were fixed in 2.5% glutaraldehyde in 0.1 M sodium cacodylate buffer, pH 7.2, at room temperature for 2 h. Subsequently, the cells underwent post-fixation in cacodylate buffer containing 1% OsO_4_, 0.8% potassium ferrocyanide and 5 mM CaCl_2_ at room temperature for 1 h. The parasites were dehydrated using an acetone series and embedded in Epon resin at 60 °C for 72 h. Ultrathin sections were obtained with an ultramicrotome (Leica). Grids containing these sections were stained with 5% uranyl acetate and lead citrate and further examined in a JEOL JEM 1400 electronic transmission microscope [26].

#### 2.4.2. Determination of Cell Volume and Mitochondrial Membrane Potential (ΔΨm)

Epimastigote forms were treated with the compounds for 24 h. To evaluate ΔΨm, the parasites were incubated for 15 min with 5 µg/mL of rhodamine (Rh) 123 (Sigma-Aldrich^®^, Barueri, Brazil). As a positive control, 10 µM carbonyl cyanide *m*-chlorophenylhydrazone (CCCP, Sigma-Aldrich^®^, Barueri, Brazil) was used [26]. To evaluate cell volume, parasites were washed twice with PBS, resuspended in the same buffer, and analyzed via flow cytometry. Actinomycin D (20 mM; Sigma-Aldrich^®^) was used as a positive control for cell volume analysis [27]. Both assays were performed using the BD Accuri^™^ C6 flow cytometer (BD Biosciences, São Paulo, Brazil), acquiring a total of 10,000 events within the region previously established as corresponding to the parasites (λexcitation/λemission: 499/680 nm for cell volume, and 515/535 nm for mitochondrial membrane potential).

#### 2.4.3. Electron Paramagnetic Resonance (EPR) Spectroscopy Analysis

The stock solution (2 mg/mL) of the spin label 5-doxyl stearic acid (5-DSA, Merck, Barueri, Brazil) was prepared in ethanol. Epimastigotes were spin-labeled as described by Alonso et al. [28] with minor modifications. Briefly, epimastigotes (5.0 × 10^7^ cells/mL) were incubated in 2 mL of LIT medium (without FBS) containing the compounds (5 × IC_50_ and 10 × IC_50_ values) for 24 h. Subsequently, the cells were washed with PBS (2500× *g*, 10 min), suspended in 50 µL of the same buffer, and each sample containing 1 x 10^8^ parasites was spin-labeled with 0.25 µL of 5-DSA ethanolic solution, and the system was gently mixed. The cells were then introduced into 1 mm capillary tubes, which were flame-sealed and centrifuged (2000× *g*, 5 min) to concentrate the parasites. EPR measurements were performed on the EPR EMX-Plus spectrometer (Bruker, Rheinstetten, Germany) using the following instrumental settings: microwave power, 2 mW; microwave frequency, 9.45 GHz; modulation frequency, 100 kHz; modulation amplitude, 1.0 G; magnetic field scan, 100 G; sweep time, 168 s; and sample temperature, 25 ± 1 °C. The total scanning range of the magnetic field in each EPR spectrum was 100 G (*x* axis), and the intensity was measured in arbitrary units (*y* axis).

#### 2.4.4. Detection of Total Reactive Oxygen Species (ROS)

Epimastigotes were treated with the compounds for 12 h, and then incubated with 10 µM 2′,7′-dichlorodihydrofluorescein diacetate (H_2_DCFDA, Sigma-Aldrich^®^, Barueri, Brazil) in the dark for 45 min. As a positive control, parasites were treated with 4 μM H_2_O_2_. Fluorescence measurements were performed in a Victor^™^ X3 multilabel plate reader (Perkin Elmer, São Paulo, Brazil), with excitation and emission wavelengths set at 488 nm and 530 nm, respectively [27].

#### 2.4.5. Determination of Lipid Peroxidation

The extent of lipid peroxidation was estimated using the fluorogenic diphenyl-1-pyrenylphosphine (DPPP, Invitrogen, São Paulo, Brazil) probe. Epimastigotes were treated with the compounds for 24 h, and subsequently, the parasites were incubated with 50 µM DPPP for 15 min at room temperature. As a positive control, 10 mM H_2_O_2_ was used. DPPP labeling was determined using a Victor^™^ X3 multilabel plate reader, with excitation and emission wavelengths set at 355 nm and 460 nm, respectively [26].

#### 2.4.6. Evaluation of Autophagic Vacuoles

Epimastigotes were incubated with the compounds, supplemented or not with wortmannin (1.6 mg/mL; Sigma-Aldrich^®^), for 24 h. Subsequently, the parasites were incubated with 5 μg/mL of the fluorescent probe monodansylcadaverine (MDC; Sigma-Aldrich^®^) in PBS for 30 min. After incubation, the cells were washed in PBS, and MDC labeling was determined using a Victor^™^ X3 multilabel plate reader with excitation and emission wavelengths set at 380 nm and 525 nm, respectively [26].

### 2.5. Statistical Analysis

The results were expressed as the mean ± standard deviation (SD) of at least three independent experiments performed in duplicate. Data analysis was conducted using GRAPHPAD PRISM version 8.0 software (GRAPHPAD Software, San Diego, CA, USA). Non-parametric data were analyzed using a one-way ANOVA test, and significant differences among means were determined using Dunnett’s test. For non-parametric data with multiple variables, a two-way ANOVA test was employed. The evaluation of intergroup statistical difference was conducted using Bonferroni’s test. In all analyses, *p* < 0.05 was considered significant.

### 2.6. In Silico Predictions of Permeability and Lipophilicity

In silico predictions of Caco-2 apparent permeability cells and lipophilicity for **BTU-1**, **BTU-2**, and **BTU-3** were assessed using the ADMETLab 2.0 platform (https://admetmesh.scbdd.com, accessed on 28 April 2023) [29].

## 3. Results and Discussion

### 3.1. BTU-1, BTU-2, and BTU-3 Inhibit the Growth of T. cruzi Epimastigotes Forms, Decreasing the Cell Volume and Inducing Significant Morphological and Ultrastructural Alterations

**Figure 1:**
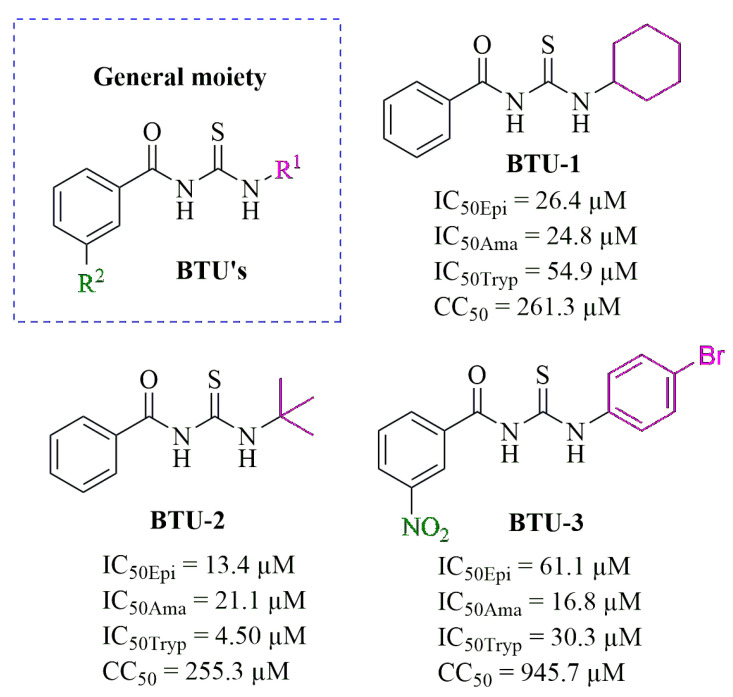
General chemical structure of benzoylthioureas (**BTUs**), along with the chemical structures of **BTU-1**, **BTU-2**, and **BTU-3.** The figure also includes the respective minimal inhibitory concentrations capable of inhibiting 50% (IC_50_) for epimastigote (Epi), amastigote (Ama), and trypomastigote (Tryp) forms of *Trypanosoma cruzi* Y strain, as well the minimal cytotoxic concentrations capable of inhibiting the viability of 50% (CC_50_) of LLC-MK2 cells. These values were reported by Pereira et al. [23].

Thiourea derivatives with a promising inhibitory effect against different species of protozoans have been described in the literature, including *Cryptosporidium hominis* [16], *Plasmodium falciparum* [14,15], *Leishmania amazonensis* [22], and *Trypanosoma* spp. [21,23]. Thus, in the search for new compounds for the treatment of CD, our research group identified that the benzoylthiourea derivatives exhibited an inhibitory effect against all developmental forms of *T. cruzi* Y strain. **BTU-1**, **BTU-2**, and **BTU-3** (Figure 1) inhibited the proliferation of epimastigotes and amastigotes, as well as the viability of trypomastigotes, maintaining non-toxic concentrations for LLC-MK2 cells after 72 h of incubation [23].

**Figure 2:**
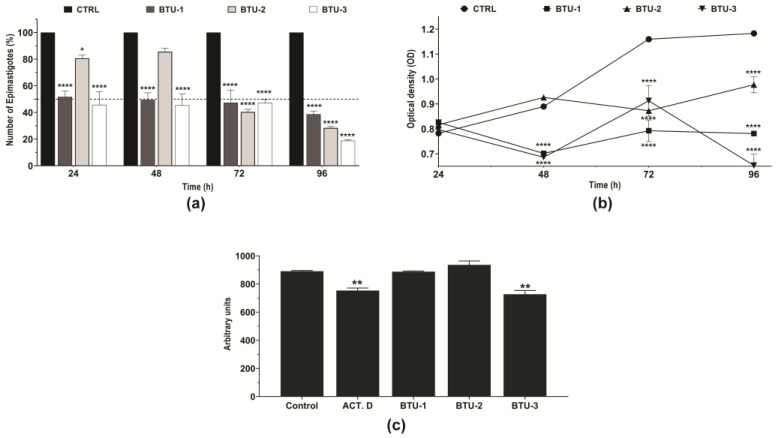
Effect of **BTU-1** (26.4 µM)**, BTU-2** (13.4 µM), and **BTU-3** (61.1 µM) on the growth, metabolic activity, and volume of epimastigotes of *Trypanosoma cruzi* Y strain. (**a**) Growth kinetics of epimastigotes in the presence of **BTU**s for 96 h were evaluated by directly counting the number of epimastigotes every 24 h. (**b**) The metabolic activity of epimastigotes was determined using resazurin dye every 24 h. (**c**) Cell volume was determined by flow cytometry after incubation in the presence of **BTU**s for 24 h. The data represent the mean ± standard deviation of three independent experiments. Actinomycin D was used as a positive control for cell volume analysis. * *p* < 0.05; ** *p* < 0.01; and **** *p* < 0.0001 compared to untreated parasites (control).

Initially, to investigate the mechanism of cell death induced by the selected **BTU**s, we analyzed the growth kinetics of epimastigotes in the presence of the derivatives at concentrations corresponding to IC_50_ values determined after 72 h of incubation [23]. The results are shown in Figure 2, in which a noticeable reduction in parasite counts was observed compared to untreated control epimastigotes. This effect persisted up to 96 h of incubation (Figure 2a). Similarly, a decrease in the metabolic activity of the parasites was observed over time compared to the untreated control (Figure 2b). These results support our previous findings. We observed that aliphatic substituents in the R1-position (Figure 1), such as cyclohexyl (**BTU-1**, IC_50_ = 26.4 µM) and *tert*-butyl (**BTU-2**, IC_50_ = 13.4 µM), showed higher activity, according to their IC_50_ values, than aromatic substituents (**BTU-3**, IC_50_ = 61.1 µM) in epimastigotes.

The presence of NO_2_ substituents is known to improve the antiprotozoal activity of synthetic substances against *T. cruzi* [30]. In fact, BZN and NFX are NO_2_-bearing compounds, acting as prodrugs. The trypanocidal effect of these drugs is activated upon the reduction of NO_2_ substituents by the parasite nitroreductase type I, leading to the formation of reactive metabolites [7,31]. The mechanism of action of NFX is mediated by unstable nitroanion radicals, which generate highly toxic reactive oxygen species (superoxide anion and hydrogen peroxide) [31]. Conversely, the reduction in the NO_2_ group of BZN generates hydroxylamine, which undergoes non-enzymatic reactions to form glyoxal dialdehyde, a highly reactive metabolite capable of forming adducts with DNA/RNA, proteins, lipids, and low-molecular-weight thiols [7,31]. Further studies are needed to evaluate the role of NO_2_ in the mechanism of action of **BTU-3.**

Flow cytometry analysis indicated a significant (*p* < 0.01) decrease (20.9%) in the cell volume of epimastigotes after 24 h of treatment with **BTU-3**, a reduction comparable to the effect induced by the positive control actinomycin D (18.1%, Figure 2c). In contrast, treatment with **BTU-1** and **BTU-2** did not cause a reduction in parasite cell volume at the tested concentration (Figure 2c).

This behavior may be linked to the structural characteristics of the compounds. Notably, **BTU-3** is phenyl-disubstituted, rendering it more lipophilic (LogP = 3.484) than **BTU-1** (LogP = 2.624) and **BTU-2** (LogP = 2.089). Consequently, the predicted Caco-2 apparent permeability (Papp) for **BTU-3** is −4.74 cm/s, while for **BTU-1** and **BTU-2**, it is −3.276, and −2.969 cm/s, respectively. These values suggest that these compounds are likely to exhibit high permeability in vivo as they can easily traverse biological membranes through passive diffusion [32,33]. Furthermore, it is well established that the cell membrane of epimastigotes contains a higher concentration of integral proteins than amastigotes and trypomastigotes [34,35]. In vitro studies have also shown that epimastigotes display a strong affinity for hydrophobic substrates while displaying limited attachment to hydrophilic substrates [36]. Therefore, the higher lipophilicity of **BTU-3** enables it to form stronger hydrophobic interactions with membrane proteins, potentially resulting in membrane damage and a greater reduction in cell volume due to cytoplasmic content leakage.

**Figure 3:**
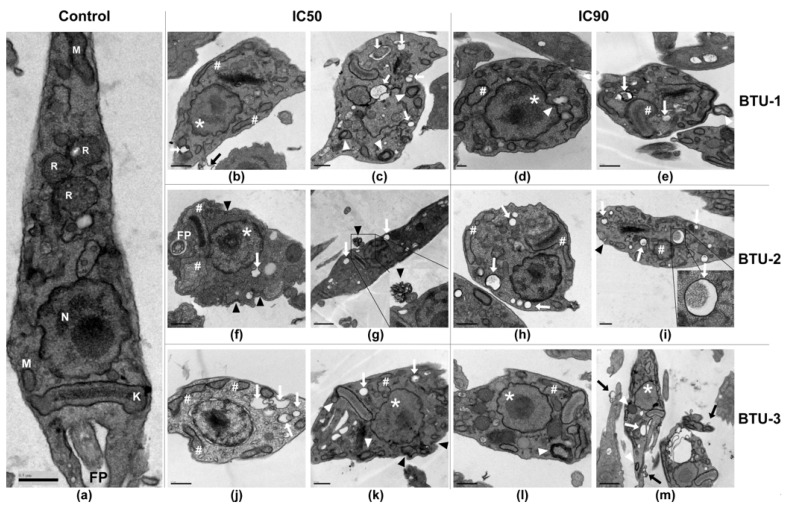
Representative transmission electron microscopy micrographs of epimastigotes of *Trypanosoma cruzi* Y strain treated with **BTU-1** (**b**–**e**), **BTU-2** (**f**–**i**), and **BTU-3** (**j**–**m**). (**a**) Untreated epimastigotes (control); (**b**,**c**,**f**,**g**,**j**,**k**) treatment with IC_50_; (**d**,**e**,**h**,**i**,**l**,**m**) treatment with IC_90_ of **BTUs** for 72 h. In the micrographs, the following structures are indicated: nucleus (N); mitochondria (M); kinetoplast (K); flagellum/flagellar pocket (FP); # mitochondrial swelling in the kinetoplast region; white arrow: vacuoles; black arrow: blebs formation in plasma membrane; black arrowhead: membrane damage and extracellular arrangement; white arrowhead: concentric membranes and myelin-like structures; * nucleus alteration. Bars: (**a**–**c**,**e**,**f**,**h**–**l**) = 0.5 µm; (**d**) = 0.2 µm; (**g**,**m**) = 1 µm.

Next, we evaluated the effect of **BTU**s on the morphology and ultrastructure of epimastigotes. Untreated epimastigotes displayed typical morphology, showing a regular electron density and presence of characteristic intracellular organelles, such as a single nucleus, the kinetoplast near the flagellar pocket, a single branched mitochondrion, and reservosomes at the posterior end (Figure 3a). However, treatment with **BTU-1**, **BTU-2**, and **BTU-3** caused remarkable morphological and ultrastructural alterations in the epimastigote forms (Figure 3b–m). After 72 h of treatment with IC_50_ of **BTU**s, nuclear disorganization, alterations in the plasma membrane inducing the formation of blebs and extracellular arrangements (Figure 3g and inset), intense cytoplasmic vacuolation with some vacuoles containing cellular residues (Figure 3i and inset), mitochondrial swelling, and formation of myelin-like structures and concentric membranes were observed. Treatment with IC_90_ of **BTU-1**, **BTU-2**, and **BTU-3** intensified the observed alterations (Figure 3d,e,h,i,l,m).

Some of these phenotypes have been identified as typical markers of cell death by the apoptosis pathway in higher eukaryotes, including nuclear disorganization, alterations in the plasma membrane, mitochondrial swelling, and decrease in cell volume [37], which have also been observed in trypanosomatids [38]. However, mitochondrial swelling, intense vacuolization, and the appearance of myelin-like figures and concentric membranes are indicative of autophagic-dependent processes [38]. Given these findings, we decided to explore the biochemical events related to the death process induced by **BTU**s in epimastigotes.

### 3.2. BTU-1, BTU-2, and BTU-3 Affect the Mitochondrial Membrane Potential (ΔΨm) of Epimastigotes

**Figure 4:**
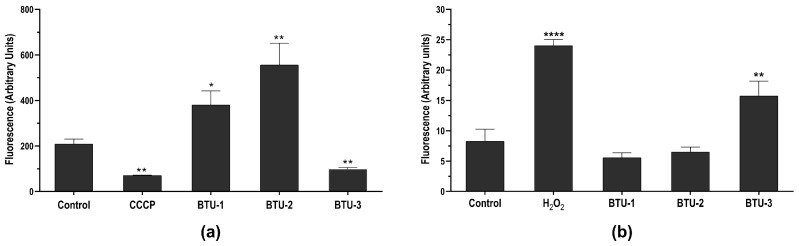
Effect of **BTU-1** (26.4 µM), **BTU-2** (13.4 µM) and **BTU-3** (61.1 µM) on mitochondrial membrane potential (**a**) and total ROS content (**b**) in epimastigotes of *Trypanosoma cruzi* Y strain. (**a**) Epimastigotes were treated with **BTU**s for 24 h and labeled with rhodamine 123. CCCP was used as a positive control. (**b**) Epimastigotes were treated with **BTU**s for 12 h and incubated with the nonfluorescent probe H_2_DCFDA. H_2_O_2_ was used as a positive control. Asterisks indicate significant differences: * *p* < 0.05; ** *p* < 0.01; and **** *p* < 0.0001 compared to untreated parasites.

Based on the TEM analysis showing mitochondrial swelling, we first assessed the impact of **BTU-1**, **BTU-2**, and **BTU-3** on the mitochondrial membrane potential (ΔΨm) using Rh123, a cationic dye that accumulates within energized mitochondria (Figure 4a). Like all trypanosomatids, *T. cruzi* presents a single mitochondrion that branches throughout the body [34,35], and the respiratory chain plays a central role in bioenergetics in epimastigotes [39]. In this study, a significant reduction (*p* < 0.01) in the total fluorescence intensity of Rh123 in epimastigotes was observed after treatment with **BTU-3** for 24 h, indicating mitochondrial membrane depolarization (Figure 4a). The loss of mitochondrial membrane potential was 53.2%. This effect was comparable to that induced by the CCCP (positive control), which caused depolarization of the inner mitochondrial membrane, resulting in reduction of 66.1% in fluorescence intensity. In contrast, treatment with **BTU-1** and **BTU-2** led to an increase in the total fluorescence intensity of Rh123, indicating mitochondrial hyperpolarization. The increase in mitochondrial membrane potential was 182.09% and 266.22% in epimastigotes treated with **BTU-1** and **BTU-2**, respectively (*p* < 0.05) (Figure 4a). A similar reduction in Rh123 fluorescence intensity has also been reported in epimastigotes of *T. cruzi* strain Y after treatment with BZN (IC_50_ value) [40].

Mitochondria play a pivotal role in controlling several events related to cell survival and death [41]. Besides energy production, the mitochondrion participates in essential cellular processes, including redox balance, stress response, and calcium homeostasis [42]. Conversely, alterations such as mitochondrial swelling and changes in membrane potential are frequently related to cell death [38]. Indeed, ΔΨm is essential to maintaining the electrochemical gradient of protons driven by the respiratory chain [41]; hence, changes in ΔΨm may represent an early event of cell death [37].

### 3.3. BTU-3, but Not BTU-1 and BTU-2, Increase Total Reactive Oxygen Species (ROS) in T. cruzi Epimastigotes

Considering the effect of benzoylthiourea derivatives on ΔΨm, the ROS levels in *T. cruzi* epimastigotes were investigated using the H_2_DCFDA probe. In the parasite, this probe is hydrolyzed by esterases, resulting in the compound dihydrochlorofluorescein (H_2_-DCF). Subsequently, this compound is converted to dichlorofluorescein (DCF), generating fluorescence in the presence of ROS [43].

**BTU-3** increased the fluorescence by around 190.44% after 24 h of treatment compared to the control group (Figure 4b), indicating an accumulation of ROS within the epimastigotes. H_2_O_2_, used as a positive control, caused a total increase of 290.32% in ROS levels. A previous study showed that BZN exhibits trypanocidal activity at concentrations that do not induce ROS production, especially superoxide anion and H_2_O_2_ [44]. This suggests that the mechanism of action of **BTU-3** may be different from that of BZN, although both induce mitochondrial depolarization. In contrast, the results showed that **BTU-1** and **BTU-2** induced a slight, but not significant (*p* > 0.05), decrease in ROS levels. This reduction may be attributed to mitochondrial hyperpolarization, which also affects mitochondrial physiology and may trigger cell death pathways.

Mitochondria serve as a primary source of ROS in most eukaryotes, with mitochondrial electron transport being the main site of production under normal physiological conditions. During mitochondrial energy metabolism, a small fraction of oxygen consumed is converted to ROS, which can act as a signal for cellular proliferation in the protozoan [45]. However, different intracellular stimuli can induce excessive ROS production, such as hypoxia, oxidative stress, and DNA damage, leading to the disruption of ATP production and the activation of cell death pathways [34,35]. Some trypanocidal compounds have been shown to affect ΔΨm, triggering the generation of high amounts of ROS in *T. cruzi* and subsequently activating parasite death through different pathways [27,46,47].

### 3.4. BTU-1, BTU-2, and BTU-3 Decrease Membrane Fluidity but Only BTU-3 Induces Lipid Peroxidation in Epimastigotes

**Figure 5:**
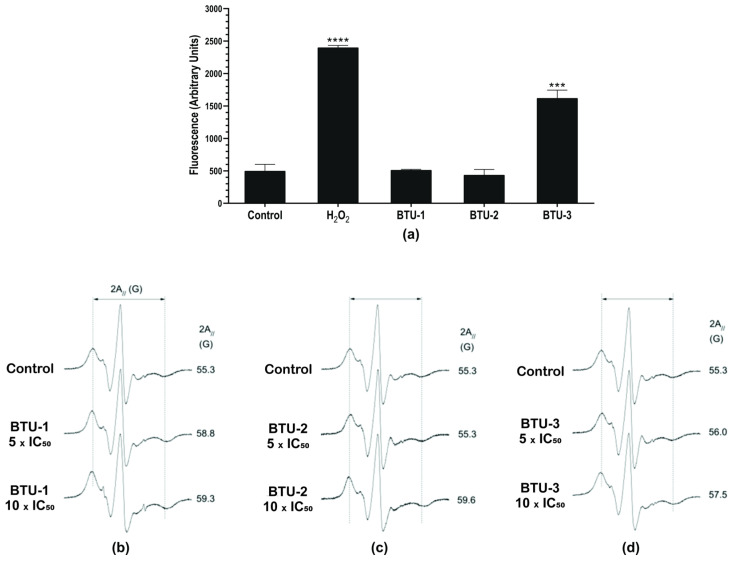
Effect of **BTU-1**, **BTU-2**, and **BTU-3** on lipid peroxidation (**a**) and membrane fluidity (**b**–**d**) in epimastigotes of *Trypanosoma cruzi* Y strain. (**a**) Epimastigotes were treated with **BTU-1** (26.4 µM), **BTU-2** (13.4 µM), or **BTU-3** (61.1 µM) for 24 h, and then labeled using the DPPP probe. H_2_O_2_ was used as a positive control. Asterisks indicate significant differences *** *p* < 0.001, **** *p* < 0.0001 compared with untreated parasites. (**b**) Representative spectra of 5-DSA spin marker inserted into membrane after treatment with different concentrations of **BTU-1** (**b**), **BTU-2** (**c**), and **BTU-3** (**d**). For each EPR spectrum, the mean value of the parameter 2A// (outer hyperfine splitting) is indicated. The 2A// value is measured directly in the EPR spectrum and is given by the magnetic field separation between the first peak and the last inverted peak. An experimental error of 0.5 G was estimated for the parameter 2A//. The increase in 2A// values in cells treated with the compounds indicates a reduction in the epimastigote membrane fluidity. EPR spectra are given by magnetic field versus absorption intensity (first derivative). On the *x*-axis, the total scan range of the magnetic field was 100 G, and on the *y*-axis, the absorption intensity is in arbitrary units.

Membrane phospholipids are among the targets of ROS in cells. Thus, we evaluated the effect of benzoylthiourea derivatives on membrane lipid peroxidation. This process results from a chain reaction of oxidative degradation of lipids, particularly affecting polyunsaturated fatty acids. Lipid peroxidation begins with the reaction between a free radical and the allylic hydrogen of unsaturated fatty acids. The generated free radical interacts with oxygen, yielding lipid peroxyl radicals that propagate lipid peroxidation. This reaction results in the formation of lipid and aldehyde hydroperoxides, including malondialdehyde (MDA) and 4-hydroxy-2′-nonenal (4HNE), which can be detected in biological samples and are used to assess oxidative stress [48]. Lipid peroxidation is considered one of the markers for oxidative stress and plays an important role in apoptosis-, ferroptosis- and autophagic-dependent cell death pathways [49]. Compatible with the ROS results, **BTU-1** and **BTU-2** had no effect on lipid peroxidation (Figure 5a). Conversely, incubation of epimastigotes with **BTU-3** induced a significant increase (1.77 times, *p* < 0.05) in the rate of the fluorescent molecule diphenyl-1-pyrenyl phosphine oxide (DPPP-O) due to its reaction with DPPP and hydroperoxides [50], indicating lipid peroxidation (Figure 5a). The positive control H_2_O_2_ induced a 2.63-fold increase in lipid peroxidation in *T. cruzi* epimastigotes.

We carried out an EPR analysis using the lipid spin label 5-DSA to gain a better understanding of alterations in the membrane of epimastigotes. This spin label is incorporated into the cell membrane, miming a lipid structure and encircling the membrane proteins. It is capable of detecting changes in molecular dynamics on both the hydrophobic surfaces of proteins and the lipid environment [51,52]. The 2A// value, a static parameter associated with the oriented distribution of the rotation of the lipid, is widely used to monitor the fluidity of the cell membrane in EPR spectroscopy analysis. Changes in this value reflect the mobility of the spin label and are represented by the separation in magnetic field units, expressed as Gauss (G) units, between the first peak and the last inverted peak of the spectra [53].

The EPR spectra in Figure 5b show that **BTU-1** decreased the mobility of the spin marker at all tested concentrations. Conversely, at 5 × IC_50_, **BTU-2** (Figure 5c) resulted in spin marker mobility similar to that of the control spectrum (2A// of ~55 G), with a slight decrease in the spin marker fluidity observed (2A// ~56.0 G) for **BTU-3** at the same concentration (Figure 5d). At 10 × IC_50_, the spin marker exhibited much lower fluidity with 2A// values increased to ~59.6 and 57.5 G for **BTU-2** (Figure 5c) and **BTU-3** (Figure 5d), respectively.

Membrane stiffness caused by certain compounds may result from the peroxidation of membrane lipids or proteins, as observed for **BTU-3**. Indeed, it has been reported that the EPR spectra of the 5-DSA marker inserted into the membrane of erythrocytes exposed to H_2_O_2_ exhibited important increases in the EPR 2A// parameter [52]. These increases were associated with oxidative processes that induced the formation of spectrin–hemoglobin crosslinks in the membrane of human erythrocytes [54]. Similarly, plasma membrane rigidity, as indicated by increases in the 2A// parameter using 5-DSA, was also detected after treatment with compounds that triggered ROS accumulation and lipid peroxidation, such as elatol in *T. cruzi* [46], and chalcone [28] and β-carboline-oxazoline [55] derivatives in *Leishmania amazonensis*. However, the membrane stiffness observed in our study could also be influenced by the presence of the benzoylthiourea derivatives (**BTU-1** and **BTU-2**) in the membrane. Alonso et al. [56] showed that the interaction of amphotericin B with membrane sterol complexes of *L. amazonensis* promastigotes caused a remarkable reduction in the 5-DSA spin label mobility, indicating membrane rigidity and/or an increase in membrane polarity. This suggests that the incorporation of specific compounds into the membrane may also contribute to alterations in membrane dynamics and fluidity.

### 3.5. BTU-1, BTU-2, and BTU-3 Increase the Formation of Autophagic Vacuoles in Epimastigotes

**Figure 6:**
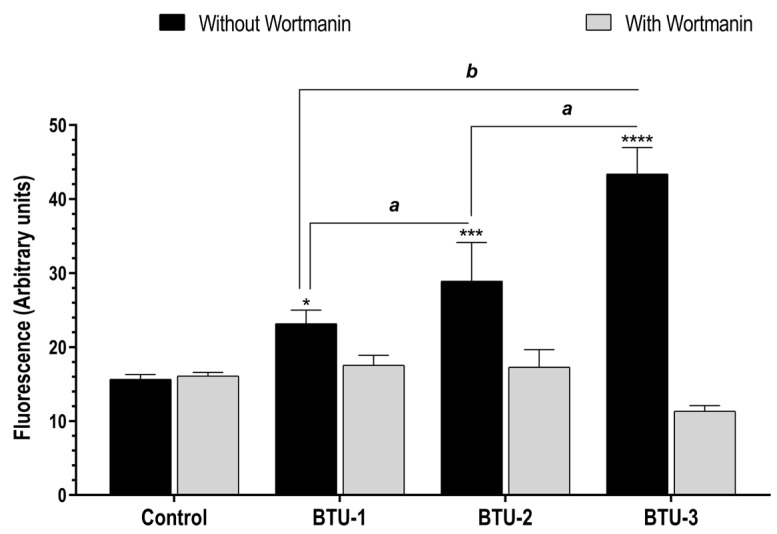
Accumulation of autophagic vacuoles in epimastigotes of *Trypanosoma cruzi* Y strain after treatment with **BTU-1** (26.4 µM), **BTU-2** (13.4 µM), or **BTU-3** (61.1 µM) supplemented or not with wortmannin. Epimastigotes were treated with the **BTU**s for 24 h and labeled with MDC probe. * *p* < 0.05, *** *p* < 0.001, **** *p* < 0.0001 compared with untreated parasites. Data not sharing a letter differ significantly between the compounds, **^a^** *p* < 0.001, **^b^** *p* < 0.0001.

In the present study, TEM analysis showed intense vacuolation and the presence of myelin-like figures in epimastigotes treated with **BTU**s, which may indicate an autophagic-dependent cell death process. In view of these data, we used the fluorescent dye MDC, which is known to accumulate in acidic autophagic vacuoles [57,58], to monitor cell death in epimastigotes. After a 24 h treatment with all **BTU**s, parasites showed an increase in the intensity of MDC fluorescence compared to the untreated control. Notably, this effect was partially prevented by preincubation with wortmannin (Figure 6), a classic inhibitor of phosphatidylinositol 3-kinase, which is known to participate in the initiation of autophagic vacuole formation [59,60]. A significant difference (*p* < 0.0001) was observed between parasites treated with **BTU-3** (3.82-fold increase) and those treated with **BTU-1** (1.32-fold) and **BTU-2** (1.67-fold) (Figure 6). These results may explain the selective reduction in cell volume observed only in response to **BTU-3** treatment (Figure 2b) under the analyzed conditions.

Autophagy is a self-digesting process of cytoplasmic components, including damaged organelles, misfolded or aggregated proteins, and oxidized molecules, achieved through the lysosomal machinery, which plays an important role in maintaining cell homeostasis [61]. Nutritional stresses are the main physiological stimulus of autophagy. Under such conditions, intracellular components are degraded to provide amino acids as an energy source for cell survival and proliferation [62]. The autophagy pathway occurs in compartments inside the cells, known as autophagic vacuoles (autophagosomes), which are morphologically characterized as double-membrane vesicles that later fuse with lysosomes [63].

In the life cycle of *T. cruzi*, autophagy is induced during metacyclogenesis (the differentiation of epimastigotes to non-replicative and infective trypomastigote forms) [64] and amastigogenesis (the differentiation of trypomastigotes to intracellular replicative amastigote forms) [65]. However, trypanocidal compounds can also induce autophagy [66,67]. For instance, dibenzylideneacetone derivates inhibited the proliferation of epimastigotes and amastigotes, and the viability of trypomastigotes of *T. cruzi* Y strain. Similar to our findings, these derivatives caused mitochondrial membrane depolarization, an increase in ROS levels and lipid peroxidation, and an accumulation of autophagic vacuoles [26].

## 4. Conclusions

The biochemical and morphological alterations induced by the benzoylthiourea derivatives **BTU-1**, **BTU-2**, and **BTU-3** collectively led to cumulative damage in mitochondrial and cell membranes, initiating a cascade of events that are incompatible with cell survival. Our data suggest that **BTU-3** induces mitochondrial membrane depolarization, resulting in increased ROS levels and plasma membrane lipid peroxidation, ultimately leading to decreased membrane fluidity and extensive cellular damage, culminating in cell death. On the other hand, **BTU-1** and **BTU-2** caused mitochondrial membrane hyperpolarization and plasma membrane stiffness, also leading to cell death. These findings indicate that the antiproliferative effect of these benzoylthiourea derivatives on epimastigotes appears to be associated with the dysfunction of multiple targets, triggering a set of phenotypes that may involve mechanisms related to autophagy-dependent pathways. It is essential to acknowledge that our study focused on the mechanisms of action of BTUs on epimastigote forms using a single concentration and treatment time in most assays. Thus, different mechanisms may be involved in the death induced by these compounds in other developmental forms of *T. cruzi*. Despite this limitation, our results indicate that these **BTU**s are promising new drug candidates for Chagas disease chemotherapy.

## Data Availability

Not applicable.
